# New method for immunoprofiling of the tumor microenvironment of cancer patients by opal multiplex quantitative immunofluorescence (IF) assay

**DOI:** 10.1186/2051-1426-3-S2-P94

**Published:** 2015-11-04

**Authors:** Nikoletta L Kallinteris, Joseph Shan, Jeffrey Meyer, Adam C Yopp, Athur Frankel, Sean R Downing, Brenda S Robertson, Cliff Hoyt, Steven King, Carlo Bifulco, Carmen Ballesteros-Merino, Bernard Fox

**Affiliations:** 1 Peregrine Pharmaceuticals, Tustin, CA, USA; 2 Department of Internal Medicine and Medical Oncology, UT Southwestern Medical Center, Dallas, TX, USA; 3 Department of Surgery, Division of Surgical Oncology, UT Southwestern Medical Center, Dallas, TX, USA; 4 Department of Hematology Oncology, UT Southwestern Medical Center, Dallas, TX, USA; 5 PerkinElmer, Hopkinton, MA, USA; 6 Earle A. Chiles Research Institute, Portland, OR, USA; 7 Providence Cancer Center, Portland, OR, USA; 8 Robert W. Franz Cancer Research Center, Portland, OR, USA

## Background

Bavituximab is a novel chimeric IgG1 monoclonal antibody targeting the membrane phospholipid, phosphatidylserine (PS), externalized on the luminal surface of endothelium in tumors, tumor cells, and tumor exosomes under stressor conditions in the tumor microenvironment. PS exposure on tumor vessels is increased by chemotherapy and irradiation, thereby amplifying the target for bavituximab. The purpose of this investigation was to develop Opal^TM^ 6-plex quantitative immunofluorescence (IF) assays ImmunoProfiling the tumor microenvironment of rectal adenocarcinoma (seven), hepatocellular carcinoma (HCC) (six), and advanced melanoma (two) patients treated with bavituximab combination treatments and to identify biomarkers that correlate immune response to patient survival.

## Methods

Opal^TM^ 6-plex quantitative IF assays utilizing Vectra Imaging System with inForm software were developed to evaluate the presence of immune infiltrates in pre- and post-treatment biopsies, or residual surgery specimen obtained from consented patients in three investigator-sponsored clinical trials with UTSW IRB approval. Formalin fixed paraffin embedded samples of patients primary tumor and post treatment, or residual surgery specimen were immuno stained with i) panel of lymphoid cell markers for CD4, CD8, PD-1, FoxP3, GranzymeB, IL-2 and DAPI, ii) panel of myeloid cell markers for CD68, iNOS, Arg-1, CD31, CD33, CD14 and DAPI, iii) panel of dendritic cell markers for PD-L1, CD11c, CD80, CD86, HLA-DR, PS, and DAPI with same species fluorescence primary antibodies labeling without cross-reactivity using tyramide signal amplification. These custom-designed assays allow single cell coexpression of multiple biomarkers in the same tissue section and can be used to quantify specific immune cells infiltration and immunomodulation of the tumor microenvironment mediated by bavituximab.

## Results

Preliminary results demonstrate reliable detection, quantification, and phenotyping of lymphocytes and monocytes present in tumor tissues obtained from consented rectal adenocarcinoma, HCC, and advanced melanoma patients treated with bavituximab combination therapies.

## Conclusions

Opal^TM^ 6-plex quantitative IF assays present a unique capability and read out assay for elucidating immunomodulation of macrophages and dendritic cells mediated by bavituximab.

Written informed consent (ICF) was obtained from all patients to collect biopsies for exploratory biomarkers research and for publication of this abstract and any accompanying images. A copy of the written consent is available for review by the editor of this journal.

